# Drift by drift: effective population size is limited by advection

**DOI:** 10.1186/1471-2148-8-235

**Published:** 2008-08-18

**Authors:** John P Wares, James M Pringle

**Affiliations:** 1 Department of Genetics, University of Georgia, Life Science Building, Athens, Georgia 30602, USA; 2 Department of Earth Sciences, University of New Hampshire, Durham, New Hampshire 03824, USA

## Abstract

**Background:**

Genetic estimates of effective population size often generate surprising results, including dramatically low ratios of effective population size to census size. This is particularly true for many marine species, and this effect has been associated with hypotheses of "sweepstakes" reproduction and selective hitchhiking.

**Results:**

Here we show that in advective environments such as oceans and rivers, the mean asymmetric transport of passively dispersed reproductive propagules will act to limit the effective population size in species with a drifting developmental stage. As advection increases, effective population size becomes decoupled from census size as the persistence of novel genetic lineages is restricted to those that arise in a small upstream portion of the species domain.

**Conclusion:**

This result leads to predictions about the maintenance of diversity in advective systems, and complements the "sweepstakes" hypothesis and other hypotheses proposed to explain cases of low allelic diversity in species with high fecundity. We describe the spatial extent of the species domain in which novel allelic diversity will be retained, thus determining how large an appropriately placed marine reserve must be to allow the persistence of endemic allelic diversity.

## Background

The relationship between genetic diversity and population size offers a number of tantalizing insights into demographic influences on evolution [1-3]. While life history characteristics of species tend to make the effective population size (N_e_) of a species much lower than the actual census size [4-6], neutral theory [7] predicts a proportional relationship between genetic diversity and N_e _[3,8]. Research has shown many cases in which N_e _as estimated from genetic markers is several orders of magnitude lower than would be predicted based on census size (N) and a species' reproductive traits [9], and it has been suggested that extremely high variance in reproductive success (the "sweepstakes" models of [1,6]) or genome-wide selective sweeps [10,11] may be causal mechanisms.

Here, using the results of Pringle and colleagues [12,13] and a simple numerical model, we quantify N_e _for populations whose dispersal is subject to persistent directional flow and find a complementary mechanism for the reduction of N_e_. We do this in a linear domain, such as a benthic population in a stream or a coastline, though the results can be easily generalized to different geometries. We find that *physical *drift in the ocean or in a stream supplements *genetic *drift as a mechanism for losing genetic diversity, and thus asymmetric dispersal – where larvae are more likely to settle to one side of their parent then another – will reduce N_e _in a given species domain. This mechanism for the reduction of N_e _will be shown to be distinct from sweepstakes models. In the sweepstakes models, N_e _is reduced by variability in reproductive success between individuals in the same region. In contrast, physical drift will be shown to reduce N_e _by creating differential reproductive success between individuals in different regions.

Previous work [e.g. [14,15]] has shown that in a linked series of populations that exchange differing numbers of migrants, the "downstream" sink population (i.e., the one that received more immigration from the "upstream" source population than the "upstream" population received from it) lost endemic alleles and eventually acquired the allele structure of the upstream population. These results suggest that the N_e _of the entire group of populations would tend to that of only the upstream population, given sufficiently large and asymmetric migration. While their work treated this effect in discrete demes, we examine a continuously distributed species along a coast or river in which there is no *a priori *partition into separate populations – a pattern that closely resembles many marine and freshwater systems. In addition, we assume that local density dependent effects limit the population and that there is no significant immigration from outside of the population being considered. We first find the region of the species domain in which the species is maintained by propagules released in that region, and not by migration from elsewhere. Then we show that it is the population of this region that acts as a source of allelic diversity and defines N_e _for the species over its entire domain. By identifying allelic retention as a spatially defined component of coastal diversity, this work has implications for the design of marine reserves. While genetic diversity has typically not been considered in the placement or size of marine reserves [16], there are clear associations between local genetic diversity and a population's resilience to stress and environmental change [17].

### An estimate of N_e _in an advective environment

In order to determine N_e_, it is necessary to divide the populations into source and sink regions. To do so, we define a "retentive population" as a demographically stable group of individuals that can persist without immigration from outside its domain. This definition is equivalent to that of Booke [18] as discussed in [19]. In coastal oceans, and other nearly one-dimensional systems such as rivers, Pringle and Wares [12] showed that a population and any alleles it contains could persist within a region if

(1) log(N_allele_) > L^2^_adv_/(2L^2^_diff_)

where N_allele _is the mean number of a given allele (or class of alleles, *sensu *[20]) in the offspring that recruit and successfully reach reproductive age per gene copy per adult per lifetime, *considering the effects of density dependence on reproductive success*. If an allele is neutral, N_allele _is equivalent to mean lifetime reproductive success per adult [12]. L_adv _is the average distance a successfully recruiting larva is moved downstream from its parent before recruiting, and L_diff _is the standard deviation of that distance for all successful recruiting larvae an adult releases. These criteria assume that kurtosis of the dispersal kernel is close to that of a Gaussian; for other kernels, a correction has been developed (Pringle et al., in review).

Allelic diversity persists within this retentive population, as the population is not supported by migration from elsewhere. We define the concept of allelic "persistence" relative to the expectation for the rate at which neutral alleles are lost or go to fixation in a finite randomly mating population [21]; with time, all allelic diversity may be transient. Alleles are considered "persistent" in a given population if they are expected to be lost or go to fixation at the rate predicted for a neutral gene in a population of that size [21,22]. As will be seen below, alleles in an advective domain that originate outside of a "retentive population" will be lost more rapidly than the neutral prediction, and will go to fixation far less often.

With advection an entire species domain cannot be a retentive population, for the criteria in equation (1) above cannot be met throughout the species range if L_adv _is not zero. At demographic equilibrium, each adult will (on average) generate one surviving offspring and thus one copy each for each copy of an allele it carries – thus the average of N_allele _over the species range is 1 for neutral alleles, which does not satisfy equation (1). Retention of some allelic diversity occurs because the reproductive success per adult is not evenly distributed spatially, and so in some places is great enough to satisfy eq. 1 [12]. One location where enhanced reproductive success must occur is the upstream edge of the model domain, for there can be no subsidy of this region by immigration from farther upstream. Byers and Pringle [13] note that if the successful reproduction at the upstream edge of the domain, and therefore N_allele _for a neutral allele, is greater than that needed to satisfy eq. (1), the population will increase at that point. This suggests that the population at the upstream edge will increase until, due to density dependent effects, the average of N_allele _over the upstream retention zone is reduced until it just satisfies eq. (1). At the upstream edge of the domain there will be a region where eq. (1) is satisfied, and novel allelic diversity can be retained. Since most larvae are transported downstream a mean distance L_adv_, this upstream region also supplies migrants to downstream regions. Thus, the upstream edge is a retentive population where alleles will only change in frequency due to stochastic drift in allele frequency and the accompanying probability of fixation.

The size and census population of the region of enhanced reproductive success, and thus N_e_, will depend on the nature of the spatial variation in habitat quality, L_adv _and L_diff_. Here we examine the case in which the habitat is spatially uniform downstream of the upstream edge of the habitat. The mean transport will move an average propagule nL_adv _downstream of its parents after n generations, while the stochastic component of transport will move the propagule a standard deviation of n^0.5^L_diff _around that point [13]. These two distances are equal after n = L^2^_diff_/L^2^_adv _generations. Substituting this expression into either of the distances defined above gives the distance L_reten _= L^2^_diff_/L_adv_, suggesting L_reten _is the fundamental length scale of this system, and is the distance over which the effects of mean and stochastic propagule transport are balanced. This suggestion is confirmed with dimensional analysis [23] by noting that "generation" is a discrete time-like dimension, and that the relations nL_adv _and n^0.5^L_diff _suggest underlying parameters with units of velocity (time/distance) and diffusivity (distance^2^/time). From these, only a single dimensionally consistent length scale can be formed, and it is L_reten_. Multiplying this distance by the carrying capacity per unit length of the environment (H_dens_) provides a scaling for N_e _comparable to that of [24]:

(2) N_e _= H_dens_L^2^_diff_/L_adv_,

and the numerical modeling described below confirms the appropriateness of this scale. (We assume that eq. (1) can be satisfied even when the population is close to its carrying capacity. When this is not true, the population is marginal at this location [13], and the estimate of N_e _will be further reduced). We expect this estimate of N_e _to be reduced relative to standard drift expectations by increasing mean propagule transport (L_adv_), and that this effect is diminished by increased stochastic transport (L_diff_). Since L_adv _and L_diff _are significantly smaller than species ranges for most coastal species [12,13,25], this value of N_e _should be much less than the census population size of the entire domain or metapopulation [as in [26]].

Downstream of the retentive population that defines N_e_, N_allele _will not satisfy (1) – and alleles are not retained – if L_adv _is non-zero. Allelic diversity in downstream regions will be set by the allelic composition of migrants from upstream. So, in an advective environment the evolution of allelic diversity in the entire population will be governed by the allelic diversity in the retentive population, and the N_e _for the entire population should approach the census population of the retentive population given by eq. (2). However, heterogeneity in abiotic (*i.e. *oceanography, temperature, salinity) as well as biotic (*i.e. *physiological responses) factors that result in an interruption or severe reduction of larval transport from upstream may lead to the formation of additional retention zones within a species' geographic range that harbor additional diversity [12,13]. As descendants of adults in the retentive populations drift downstream in large species domains, they may acquire additional allelic diversity through mutation. It will be argued below that for most realistic population and dispersal parameters this latter effect is small.

## Methods & results

To test these ideas, we use a simple numerical model, similar to those used by Pringle and colleagues [12,13]. In our model, a haploid semelparous individual produces a fixed number of larvae that disperse on average a distance L_adv _downstream, with standard deviation of L_diff _with a Gaussian dispersal kernel [27] or with Laplace's distribution. Density dependence exists because if more than one propagule recruits to the same location, one is randomly chosen to survive. The model domain is finite, and any propagules that leave the domain die. The results shown below are computed in a model with a low population carrying capacity per unit length (of order 1 individual/km), due to limited computational resources. However, the validity of these results is not sensitive to the magnitude of this parameter.

**Figure 1:**
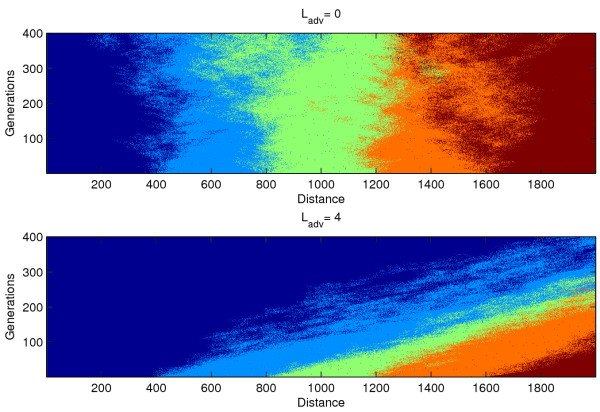
**In both (A) and (B), the domain is initialized with haploid adults containing 5 different alleles, each geographically isolated to 1/5 of the domain, and each adult colored according to its allelic composition.** The model is run for 400 generations. In (A), L_adv _= 0 km and L_diff _= 10 km. The allelic composition diffuses isotropically away from initial positions, and no allele is favored over others. In (B), L_adv _= 4, so larvae preferentially disperse towards positive x (to the right) and the upstream allele quickly dominates the entire domain. L_reten _in (B) is 25 km.

To illustrate how advection reduces genetic diversity in a population, two domains are initialized with five different alleles each in different parts of the domain in the numerical model (figure 1). In one domain, mean transport of larvae L_adv _is zero; in the other it is 4 km/generation to the right. In both, the stochastic component of larval transport L_diff _is 10 km/generation. In the case with no mean larval transport, all genetic diversity is retained. However, when there is mean larval transport, only the upstream allele persists and the other alleles are lost downstream, for only the upstream allele begins in the retentive region that lies within L_reten _= 25 km of the upstream edge of the domain.

**Figure 2:**
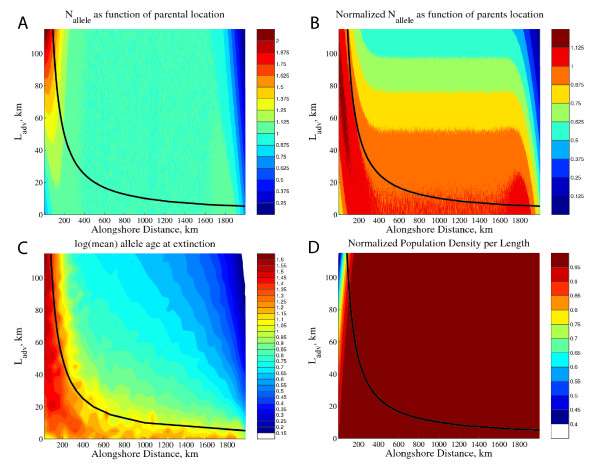
**(A) N_allele _for a 1-dimensional ocean with a mean current from left to right as a function of the alongshore distance and the mean larval transport distance, L_adv_.** The heavy black line represents the width of the retention zone L_reten _= L^2^_diff_/L_adv _from the upstream edge of the domain. (B) N_allele _normalized by the critical value of N_allele _needed to allow retention, as given by eq. (1). (C) The logarithm of average persistence time of a novel allele in generations as a function of the location where the allele first appeared. (D) The population density per length of the domain, normalized by the carrying capacity. The stochastic component of larval transport, L_diff_, is 100 km, the carrying capacity of the domain is 1 individual/km, and the domain is 2048 km in size.

A second numerical experiment illustrates how the presence of directional larval dispersal changes the spatial structure of the population system. In these model runs, L_diff _is fixed to 100 km and L_adv _is varied from 0 to 116 km. There is a mutation rate μ = 10^-3 ^such that larvae randomly carry a new allele with this frequency (a smaller, more realistic μ does not change the results, but dramatically increases computation time). In these model runs, N_allele _is uniform in the interior and small near the edges when L_adv _is zero, but as L_adv _increases, N_allele _becomes largest near the upstream edge of the species domain, and is one in the interior of the model domain (figure 2A). Examining N_allele _divided by the value that just satisfies eq. (1) (figure 2B), we find that N_allele _just satisfies eq. (1) in the retention zone that lies within L_reten _from the upstream edge of the domain, and does not elsewhere. In the model, time to fixation or extinction of all novel alleles is tracked as a function of their origin. As discussed above, enhanced reproductive success within a distance L_reten _from the upstream edge allows novel alleles to persist longer in the upstream retentive population, for a time appropriate to N_e _as given by Eq. (1), while those in downstream regions are lost much more quickly (figure 2C). The region in which novel alleles are retained, and the density of these upstream regions, decreases in size as L_adv _increases (figure 2D), as predicted in the expression for L_reten_.

**Figure 3:**
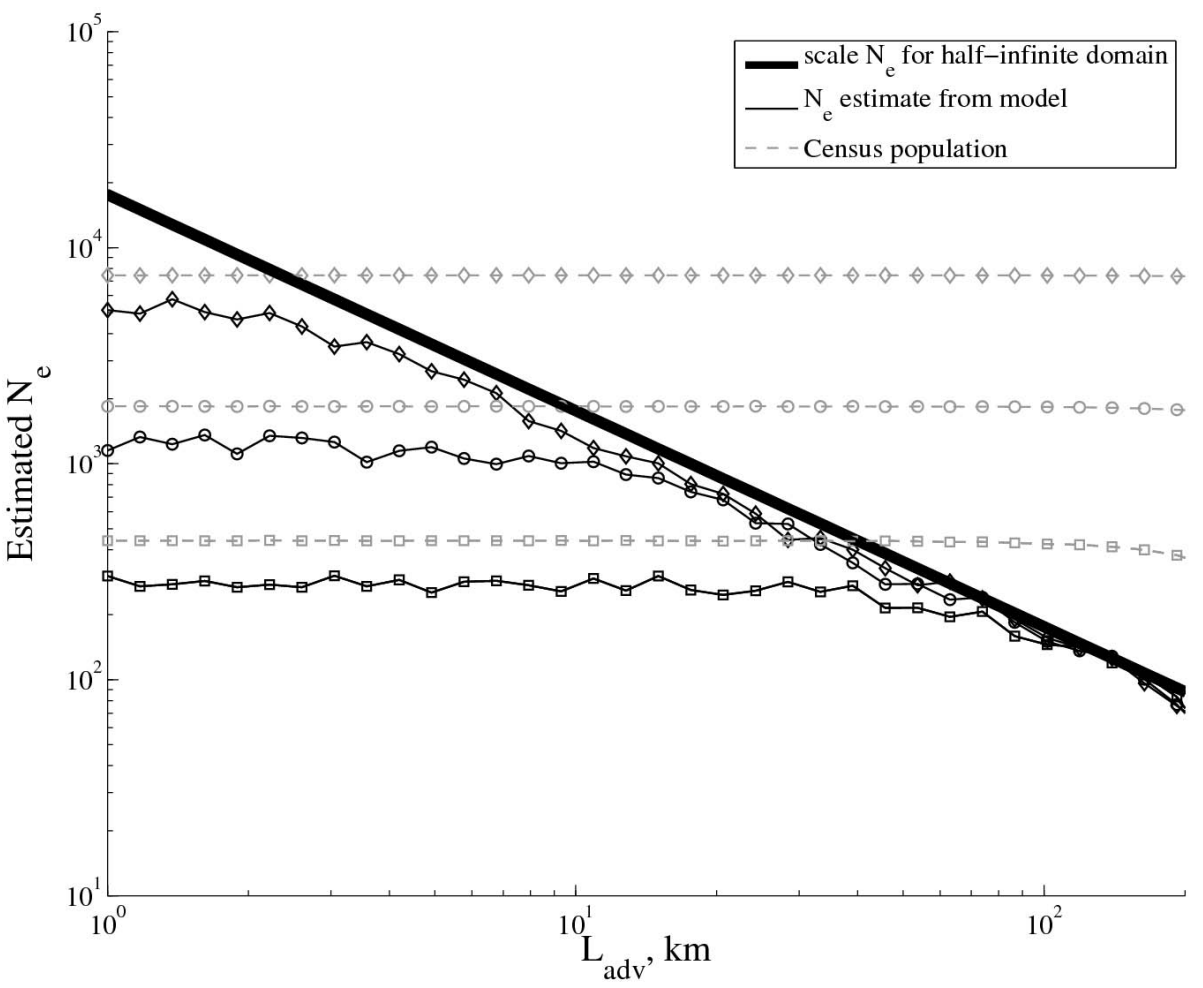
**(Thick Black Line) Estimate of N_e _from equation (2).** (Dashed Thin Lines) Census population in domain. The squares (□) are for a domain 1024 km in size, the circles (○) are for a domain 4096 km in size, and the diamonds (◇) for a domain 16384 km in size. (Solid Lines) Estimates of N_e _from a numerical model with varying L_adv _and a constant L_diff _of 200 km. For the purposes of simulation, the carrying capacity of the domain is about 0.5 individuals per kilometer.

To determine N_e _as a function of L_adv _and L_diff_, we calculate the inbreeding effective population size N_e _[28] given the mean lifetime of a novel allele in the system. We initialize the model with two alleles, each randomly distributed and each comprising 50% of the population. The neutral time to fixation in such a model will be 2.7 N_e _[21], and so we estimate N_e _from the average fixation time of 100 model runs. In Figure 3, we run the model in three domains of sizes L_domain _= 10^3^, 4 × 10^3^, and 1.6 × 10^4 ^km. In each domain, we fix L_diff _to 200 km, and vary L_adv _from 0 to 110km, and compare the estimated N_e _from (2) to the estimation from fixation time in an upstream region of the model L_reten _in size. Once there is fixation in this upstream region, the allele fixes rapidly in the rest of the species domain in approximately L_domain_/L_adv _generations. When the size of the domain is less than L_reten _in extent, N_e _is limited to the population census size (figure 3). Thus when L_adv _is small, N_e _is nearly equal to the census population of the entire population, though somewhat smaller due to loss of larvae from the edges of the domain caused by stochastic larval transport. When the domain size is greater than L_reten_, eq. (2) captures the variability of N_e _with L_adv _very well, capturing the several order of magnitude decline in N_e _with increasing L_adv_. As mentioned above, the estimate of N_e _from (2) is, for most values of L_adv_, very much smaller than – and not dependent upon – the census population size. When the model is re-run with Laplace's dispersal kernel, the results shown in Figure 3 remain unchanged (not shown), suggesting that these results are not very sensitive to the kurtosis of the dispersal kernel.

## Discussion

The concept of "effective population size" is typically used as a numerical trait of a population more than as a descriptor of biological reality [26]. But N_e _is intended to reflect the number of individuals that contribute to the evolutionary potential of a species [29]; in advective environments, we have shown that this contribution is driven mostly by a small upstream portion of a species geographic range. Given selective neutrality of the genes being studied in a standard population genetics survey, it is generally assumed that the number of successful offspring an individual will have is independent of its genotypic state or geographic location [3]. Other results have suggested that for some species, habitat may be structured by varying quality that will determine the distribution of offspring numbers [6], *i.e. *a "nest-site" model [3]. In these cases, however, it is typically assumed that location itself (and thus the potential for lower variance in reproductive success) is not heritable. In the case of populations under mean advection, however, the upstream retention zone will represent a heritable component of reproductive success for any larvae spawned there because a small fraction of these larvae retain their parents' geographic reproductive advantage.

The attention given to estimating N_e _in natural populations has recently been focused on a number of demographic causes for reduced N_e_/N ratios [6,26,30]. Here we show a significant environmental interaction that can strongly affect diversity in continuously distributed species. While overall allelic diversity in the species' domain will likely include a large number of potentially transient alleles that form in downstream regions at a rate 2 N_e_μ (or N_e_μ for haploid markers), in an advective environment most of these are more quickly lost due to advection (a time in generations of about L_domain_/L_adv_) than to stochastic genetic drift (Figure 2). The retention of diversity is thus going to be related to the size of demographically stable and retentive populations such as the upstream edge of the domain, a size that is specific to a species' reproductive and larval dispersal traits. As L_adv _increases, this source region becomes smaller, and with uniform density of individuals reduces N_e _concomitantly.

Thus, different species with distinct larval dispersal traits can have distinct N_e_/N ratios in the same region, all else being equal. This mechanism does not hinge on the reproductive "sweepstakes" between individuals at the same location – instead, it is an effect of the differential reproductive success of individuals from different regions, and the effects of mean larval transport. Mean larval transport, L_adv_, can change from generation to generation [13], and therefore the size of the retention region can vary from generation to generation. This will produce a fluctuating N_e _from generation to generation, and years of especially strong mean advection could reduce net diversity. Thus N_e _can be reduced not only by year-to-year variation in reproductive success, but also by inter-annual changes in the physical environment that affect larval dispersal.

A growing body of literature attempts to link patterns of genetic diversity with patterns of biodiversity, for the purposes of elucidating the mechanisms underlying broad-scale biogeographic structure and for conservation-focused predictions [31,32]. However, in strongly advective environments the link between population genetic structure and community structure may be tenuous because what appears to be panmixia – extensive, range-wide gene flow – may instead represent an extended source-sink metacommunity [26]. Our predictions suggest that in populations whose dispersal is subject to strong advection (*e.g.*, high dispersal from the upstream populations to downstream) N_e _will be more disassociated from actual census size than for populations less affected by advection.

To test this prediction, one might imagine comparing species with very different dispersal strategies, or comparing the same or similar species in two locations with different dispersal conditions. However, while classical population genetics predicts elevated diversity *across *populations of low-dispersal species, recent isotropic descriptions of metapopulation structure [26] show that due to unequal contribution of some populations to subsequent generations, limited dispersal can actually reduce N_e_. Metapopulation structure in general may bias the measurement of N_e _and gene flow measures [33], and Foltz [34] suggests a strong role of purifying selection and/or local adaptation in limiting diversity in dispersal-limited species. Altogether, these factors may confound comparisons of Ne/N ratios among distantly related taxa; thus it may be more appropriate to make intraspecific or sister-taxon comparisons, in which closely-related equilibrium (e.g. not introduced or range-expanded) populations exist on a "strong-advection" coast and a "weak-advection" coast. Such a comparison can be made for species with pelagic larvae that are distributed on both the Pacific coast of the Baja California peninsula (strong advection) and in the semi-enclosed Gulf of California (weak advection). Our work would predict higher diversity in the weak advection Gulf of California region; given six appropriate studies, four support this hypothesis [35-38], one is equivocal but at least some population samples in the Gulf have higher diversity [39], and in one case the Gulf sister species is apparently less diverse [40].

## Conclusion

Overall, some of the lowest N_e_/N ratios observed are for species with broad dispersal potential in regions where ocean currents would be expected to generate a large L_adv _[6,41]. While a species' fecundity may be associated with dispersal potential in marine organisms [42], fecundity alone is not predictive of Ne/N [43] – suggesting that other factors, including advection, may be involved in the relationship between N_e _and actual census size. In the end, any mechanism that increases the variance in reproductive success among individuals, whether due to stochastic, biological, or spatial processes, will reduce genetic variation in a species [28]. Here we argue that one of many processes that must be considered when analyzing the genetic diversity of coastal species is the interaction between a species and its dispersal. Dispersal must be considered in the persistent discussion of N_e_/N ratios (*e.g.*, [11]) if there is to be a better empirical understanding of how variation is maintained in natural populations, and for management and conservation questions [44]. Our work explicitly defines the size of domain that will be evolutionarily important and is relevant to marine reserve design, in that if a reserve is larger than this region of size L_reten _it will also be protecting 'sink' regions; if it is smaller than this length, reserve size will be a limiting factor on total genetic diversity.

## Authors' contributions

This work was developed as equal-authorship collaboration between JPW, who conceived of the study and drafted the manuscript, and JMP who developed the numerical simulations and scalings and coordinated the results, and helped to draft the manuscript. All authors read and approved the final manuscript.
